# *POLE* mutations in endometrial carcinoma: Clinical and genomic landscape from a large prospective single‐center cohort

**DOI:** 10.1002/cncr.35731

**Published:** 2025-01-25

**Authors:** Camilla Nero, Rita Trozzi, Federica Persiani, Simone Rossi, Luca Mastrantoni, Simona Duranti, Floriana Camarda, Ilenia Marino, Luciano Giacò, Tina Pasciuto, Maria De Bonis, Martina Rinelli, Emanuele Perrone, Flavia Giacomini, Domenica Lorusso, Alessia Piermattei, Gianfranco Zannoni, Francesco Fanfani, Giovanni Scambia, Angelo Minucci

**Affiliations:** ^1^ Unit of Oncological Gynecology Department of Women, Children and Public Health Sciences Fondazione Policlinico Universitario Agostino Gemelli IRCCS Rome Italy; ^2^ Università Cattolica del Sacro Cuore Rome Italy; ^3^ Bioinformatics Research Core Facility Gemelli Science and Technology Park (G‐STeP) Fondazione Policlinico Universitario Agostino Gemelli IRCCS Rome Italy; ^4^ European School of Molecular Medicine (SEMM) Milan Italy; ^5^ University of Milan Milan Italy; ^6^ Medical Oncology Università Cattolica del Sacro Cuore Rome Italy; ^7^ Scientific Directorate Fondazione Policlinico Universitario Agostino Gemelli IRCCS Rome Italy; ^8^ Epidemiology and Biostatistics Facility, G‐SteP Fondazione Policlinico Universitario Agostino Gemelli IRCCS Rome Italy; ^9^ Departmental Unit of Molecular and Genomic Diagnostics Genomics Core Facility G‐STeP Fondazione Policlinico Universitario Agostino Gemelli IRCCS Rome Italy; ^10^ Gynecologic Oncology Unit Humanitas San Pio X, Humanitas University Milan Italy; ^11^ Gynecopathology and Breast Pathology Unit Department of Women, Children and Public Health Sciences Fondazione Policlinico Universitario Agostino Gemelli IRCCS Rome Italy

**Keywords:** adjuvant therapy, comprehensive genomic profiling, DNA polymerase epsilon (POLE), endometrial cancer, molecular classification, POLE hotspots, POLE multiclassifier

## Abstract

**Background:**

To date, 11 DNA polymerase epsilon (*POLE*) pathogenic variants have been declared “hotspot” mutations. Patients with endometrial cancer (EC) characterized by *POLE* hotspot mutations (*POLE*mut) have exceptional survival outcomes. Whereas international guidelines encourage deescalation of adjuvant treatment in early‐stage *POLE*mut EC, data regarding safety in *POLE*mut patients with unfavorable characteristics are still under investigation. On the other hand, the spread of comprehensive genome profiling programs has underscored the need to interpret *POLE* variants not considered to be hotspots.

**Methods:**

This study provides a comprehensive analysis of 596 sequenced patients with EC. The genomic landscape of *POLE*mut EC was compared with cases harboring nonhotspot *POLE* mutations within the exonuclease domain. Additionally, the genomic characteristics of multiple classifiers, as well as those exhibiting unfavorable histopathological and clinical features, were examined.

**Results:**

No significant genomic differences were observed among patients with *POLE*mut EC when comparing multiple classifiers to not‐multiple classifiers or those with unfavorable clinical features. However, the tumor mutational burden differed in both comparisons, whereas the percentage of C>G mutations only differed in the comparison based on clinical features. Specific *POLE* mutations, even if not considered to be hotspots, have genomic features comparable to *POLE*mut.

**Conclusions:**

The present findings confirm the absence of significant genomic differences among *POLE*mut patients regardless of multiple‐classifier status or association with high‐risk clinical features. Prognostic data will be essential to elucidate the clinical significance of *POLE* mutations not classified as hotspots that exhibit genomic characteristics similar to those in *POLE*mut patients.

## Abstract 2

This comprehensive genomic analysis of 596 patients with endometrial cancer investigated the characteristics of DNA polymerase epsilon (*POLE*) mutations. The results revealed no significant genomic differences besides tumor mutational burden values among *POLE*mut patients regardless of multiple‐classifier status or association with high‐risk clinical features. Additionally, some *POLE* mutations not classified as hotspots but within the exonuclease domain displayed genomic characteristics akin to those of *POLE*mut patients.

## INTRODUCTION

International guidelines have increasingly integrated molecular risk assessment into the management of patients with endometrial cancer (EC), with the aim of better tailored therapeutic strategies.^1^ Within the four existing molecular subgroups, less than 10% of patients with EC harbor pathogenic variants within the exonuclease domain (EDM) of DNA polymerase epsilon (*POLE*).^2^ *POLE* pathogenic variants are characterized by unique features, including a notable increase in C>A substitutions, which account for more than 20% of alterations, a relatively low frequency of small insertion and deletion variants (indels), and an exceptionally high tumor mutational burden (TMB; >100 mutations/megabase [Mut/Mb]).^3^ To date, 11 *POLE* pathogenic variants have been declared “hotspot” mutations. Five of these were highlighted in a TCGA (The Cancer Genome Atlas) article^3^ as the most frequently associated, with an ultramutated phenotype (P286R, V411L, S297F, A456P, and S459F), whereas the remaining six were identified via a pragmatic scoring system developed by León‐Castillo et al.^4^ This system evaluated the TMB, specific nucleotide changes (C>A, T>G, and C>G), indel proportions, and results from six in silico tools applied to whole‐exome sequencing.

Patients with EC with one of the *POLE* hotspot mutations exhibit remarkably favorable survival outcomes (>98% 5‐year survival) despite unfavorable histomorphological features such as high grading, deep myometrial invasion, and/or lymphovascular invasion.^5^ For these reasons, *POLE* status is now included in the latest FIGO (International Federation of Gynecology and Obstetrics) staging for EC, and adjuvant treatment is not recommended for FIGO stage I–II *POLE*mut patients.^6^ However, this new integrated risk classification model is raising concerns. The excellent prognosis associated with *POLE*mut EC is based on a retrospective series and secondary analysis of clinical trials. According to the guidelines in place at the time, most of these patients received adjuvant treatment, yet some still relapsed and even died from the disease.^5^,^7^ Referral and academic centers have been implementing comprehensive cancer genome profiling as an upfront test for oncological patients, which grappled with the complexities of data interpretation and unexpected findings.^8^ In particular, interpreting somatic *POLE* mutations within EDM that are not recognized as hotspots, along with *POLE* hotspot mutations exhibiting atypical molecular features (such as a low TMB or unfavorable pathological characteristics), can be particularly challenging.

With the objective of better delineating comprehensive features of cases with *POLE* mutations, hotspots, and not, we here present a genomic, pathological, and clinical characterization of an unselected series of prospectively clinically sequenced patients with EC from a large referral center.

## MATERIALS AND METHODS

At Fondazione Policlinico Universitario Agostino Gemelli IRCCS, patients with specific solid tumors are offered a tumor‐only targeted next‐generation sequencing panel as part of an institutional comprehensive cancer genome profiling (CGP) program (ClinicalTrials.gov identifier NCT06020625; protocol FPG500).^9^

This program adheres to the guidelines outlined in the Declaration of Helsinki, and received approval from the Fondazione Policlinico Universitario Agostino Gemelli IRCCS ethical committee (protocol U 00194/23; ID number 3837). Before participation, all patients provided informed consent.

### Data collection

Demographic and clinical data of enrolled patients were collected via a customized electronic case report form (eCRF) by the review of medical records. The eCRF was developed with REDCap (https://redcap‐irccs.policlinicogemelli.it). This web application is fully compliant with EU guidelines for data protection and management (General Data Protection Regulation 2016/679).

### Histopathological data

Histopathological data were obtained from pathology reports generated by the gynecologic pathology unit, which operates with a standardized diagnostic approach (https://www.mayocliniclabs.com/test‐catalog/overview/35466). The presence and extent of lymphovascular space invasion (LVSI) were scored as absent, focal, or substantial. Additionally, the results of the immunohistochemistry (IHC) analysis of the p53 and DNA mismatch repair (MMR) proteins MLH1, MSH2, MSH6, and PMS2 were recorded (see Supporting Information S1: Supplementary Materials).

### Genomic data analysis

Details on genomic methodologies have been previously published, and are available in Supporting Information S1: Supplementary Materials.^10^ Briefly, the TruSight Oncology 500 (TSO 500) multigene panel (Illumina, San Diego, California) was applied to samples containing more than 20% tumor cells as determined by a review conducted by two gynecologic pathologists (A.P. and G.Z.). This panel can detect substitutions, insertion and deletion alterations, and copy number alterations across 523 genes, as well as selected gene rearrangements and TMB. Microsatellite instability (MSI) status was determined by analyzing 130 homopolymer repeat loci (10–20 base pairs) with a minimum coverage of 60 full‐spanning reads. The TMB was considered high if there were ≥10 Mut/Mb.

### Molecular subtyping

Molecular subtype assignment was conducted by integrating sequencing data with an IHC approach according to European Society for Medical Oncology guidelines.^11^,^12^ Patients with EC were classified as follows: (1) *POLE*mut, based on the presence of one of the *POLE* mutations included in the list of 11 pathogenic variants^4^; (2) MMR deficient (MMRd), determined by IHC; (3) p53 abnormal (p53abn), identified via IHC; and (4) nonspecific molecular profile (NSMP), characterized by the absence of the defining features of the previous molecular subtypes.

Furthermore, with respect to *POLE* status, two subgroups were established for the purposes of this analysis:

Group A: *POLE* variants classified as hotspots, as described by León‐Castillo et al.,^4^ which corresponded to *POLE*mut patients.
Group B: *POLE* nonhotspot variants within the EDM, which did not result in *POLE*mut patients. Patients within group A who carried a concomitant p53abn and/or the absence of MMR proteins at immunostaining were defined as multiple classifier (MC).^13^

### TCGA feature analysis and mutational signatures

The percentage of TCGA features (base substitutions and indels) was calculated with the Maftools and MutationalPatterns packages.^14^

Mutational signatures were inferred from TSO 500 HT data with single‐nucleotide variants (SNVs) from a filtered mutation annotation format. The SigProfilerAssignment package^15^ was used to deconstruct the mutation spectra in each tumor and compare their mutational profiles with reference signatures from the Catalog of Somatic Mutations in Cancer (COSMIC), version 3.4, database. The focus was on signatures commonly associated with proofreading defects of polymerases in samples with intact MMR machinery.

### In silico prediction tools

To evaluate the functional status of *POLE* in group B, we used PolyPhen and Sorting Intolerant From Tolerant, in silico prediction tools for nonsynonymous variants based on sequence homology. Moreover, we used the Rare Exome Variant Ensemble Learner for predicting the pathogenicity of missense variants on the basis of a combination of scores from 13 individual tools,^16^ and MetaRNN, a pathogenicity prediction model for human nonsynonymous SNVs and nonframeshift indels, which integrates information from 28 high‐level annotation scores and produces an ensemble prediction model via a deep recurrent neural network.^17^

### Statistical analysis sample size

The sample size of this retrospective study was not hypotheses driven but rather based on a time window (March 2022 to December 2023). All the analyses were descriptive, and the data were summarized with absolute counts and percentages if related to categorical items and mean and standard deviation ranges when considering quantitative variables. Differences between groups were investigated with the Mann–Whitney *U* test or Kruskal–Wallis test as appropriate. Principal component analysis (PCA) was performed to visually inspect groups according to TCGA features. In brief, the first two PCs explained 75% of the variance; C>A and TMB showed higher loading for PC1, whereas C>G and indels showed higher loading for PC2.

## RESULTS

Of 3125 patients enrolled in the institutional CGP program (FPG500) from March 1, 2022, to December 31, 2023, 695 were EC cases (Figure S1). In all cases, the primary tumor specimen was sequenced after a complete surgical staging including sentinel node mapping.^1^ Of these, 596 patients with EC had complete clinical, IHC, and genomic data, and were therefore included in the present study. Ten percent were *POLE*mut, 34% were MMRd, 13% were p53abn, and 42% were NSMP. Clinicopathological characteristics are shown in Table S1. The majority (62.4%) of the entire population had a low‐grade endometrioid histological subtype and a uterine‐confined disease. Mutational and clinical characteristics of the entire population are shown in Figure S2. Median follow‐up was 19.6 months (95% confidence interval [CI], 18.8–20.8 months), 31 of 596 patients (5.2%) experienced recurrence or progression during treatment, and 14 of 596 patients (2.3%) died of the disease (0% disease‐ and 0% cancer‐specific deaths in group A patients).

### Analysis of group A

Clinical characteristics of this group are reported in Table 1. Median age was 57 years. Most of the patients had grade 1 or 2 (44.3%) or grade 3 (32.8%) endometrioid histotypes, followed by mixed, clear cell, and serous. Twenty‐three percent of cases had substantial LVSI. Four patients had an advanced stage according to both 2009 and 2023 FIGO staging. Ten patients were negative for both estrogen and progesterone receptors (ER and PR).

**TABLE 1:** Clinical and pathological characteristics of group A.

Characteristics	Hotspot *POLE* variant
	*N* = 61
Age at diagnosis, mean (range), years	55 (31–88)
Histotype, No. (%)	
Endometrioid G1 and G2	27 (44.3)
Endometrioid G3	20 (32.8)
Serous	2 (3.3)
Clear cell	3 (4.9)
Mixed	6 (9.8)
Dedifferentiated	1 (1.6)
Carcinosarcoma	2 (3.3)
LVSI, No. (%)	
Negative	41 (67.2)
Focal	6 (9.8)
Substantial	14 (23.0)
2009 FIGO pathological stage, No. (%)	
IA	36 (59.0)
IB	17 (27.9)
II	4 (6.6)
IIIA	1 (1.6)
IIIC1	2 (3.3)
IVB	1 (1.6)
2023 FIGO pathological stage, No. (%)	
IAm_*POLE*mut_	57 (93.4)
IIIA1	1 (1.6)
IIIC1i	2 (3.3)
IVC	1 (1.6)
Multiple classifier, *n* of *N* (%)	
*POLE*mut‐MMRd	9 of 16 (56.3)
*POLE*mut‐p53abn	4 of 16 (25.0)
*POLE*mut‐MMRd‐p53abn	3 of 16 (18.8)

Abbreviations: FIGO, International Federation of Gynecology and Obstetrics; G, grade; LVSI, lymphovascular space invasion; MMRd, mismatch repair deficient; p53abn, p53 abnormal; *POLE*, DNA polymerase epsilon.

**FIGURE 1:**
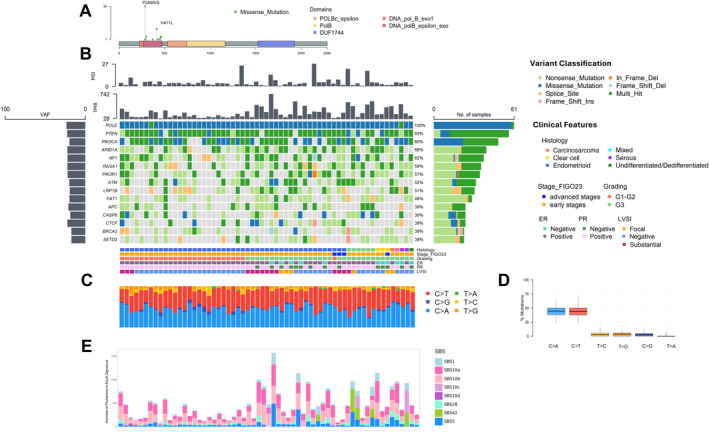
Integrative genomic and clinical characterization of patients in group A. (A) Lollipop plot representing mutation distribution in the POLE gene. Missense mutations and functional domains are reported. Most common mutations were P286R/S and V411L. (B) Oncoplot displaying variants in the most frequently mutated genes and clinical features. Bar chart on the left represents the mean VAF. (C–D) Single nucleotides substitution types. (C) Percentage of single nucleotide substitutions per sample and (D) Box plot illustrating single nucleotide substitutions frequencies for different substitution types. Points outside the whiskers represent outliers. (E) COSMIC mutational signature decomposition. Bar plot displaying the contribution of mutational signatures to each sample. Different colors represent distinct signatures. COSMIC, catalogue of somatic mutations in cancer; ER, estrogen receptor; FIGO, International Federation of Gynecology and Obstetrics; G, grade; LVSI, lymphovascular space invasion; MSI, microsatellite instability; *POLE*, DNA polymerase epsilon; PR, progesterone receptor; TMB, tumor mutational burden; VAF, variant allele frequency.

All of group A displayed missense mutations, the most frequent being P286R (36 patients; 59.01%) and V411L (12 patients; 19.67%) (Figure 1A; Table S2). Two patients, beyond the *POLE* hotspot mutation, displayed a concomitant *POLE* mutation within the EDM. The most frequently comutated genes with *POLE* were *PTEN* (93%), *PIK3CA* (80%), *ARID1A* (66%), *NF1* (62%), and *RASA1* (59%). Median TMB was 150.8 Mut/Mb (Figure 1B).

Additionally, we investigated the other TCGA features associated with mutated phenotypes, such as the percentage of C>A, T>G, and C>G nucleotide substitutions and the percentage of indels. Median values were 1.78% for indels, 44.44% for C>A, 2.85% for C>G, and 3.70% for T>G (Figure 1C,D). We then compared the TCGA features with the other molecular subgroups. As expected, the TMB of group A was higher than that of the other groups (*p* < .001). Furthermore, post hoc tests showed the highest values in group A for C>A (*p* < .001) and T>G (*p* < .001) and significantly lower values for indels (*p* < .001) and C>G (*p* < .001) compared with the other groups.

All cases, except for two, displayed a contribution of COSMIC mutational signature 10, as expected (Figure 1E). Both cases had unfavorable clinical and histopathological characteristics and were MC. The first patient (*POLE* mutation V411L) was affected by a mixed EC, with serous (80%) and endometrioid (20%) grade 3 components and substantial LVSI. She displayed 17.77% indels, 25.00% C>A, 0.00% C>G, and 0.00% T>G. The second patient (*POLE* mutation D368Y) was affected by grade 3 endometrioid EC, and showed 60.00% indels, 30.00% C>A, 0.00% C>G, and 0.00% T>G. Both received adjuvant radiotherapy as a result of incomplete surgical staging, and per the last follow‐up (3.3 and 0.8 months, respectively), they are alive and free of disease.

In the PCA of the entire cohort, group A emerged as a cluster along the PC1, whereas MMRd patients were separated from p53abn and NSMP along the PC2.

**FIGURE 2:**
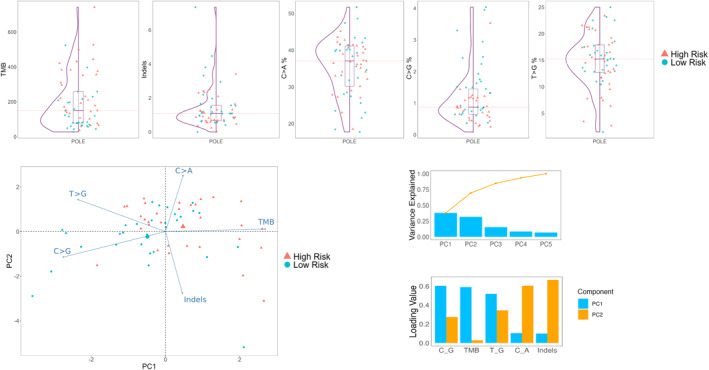
Comparison and principal component analysis of The Cancer Genome Atlas parameters across group A. Top row, from left to right: Distribution of TMB, Indels, C>A substitutions and T>G substitutions: between high‐risk (red triangles) and low‐risk (blue circles) samples within the POLE group. Bottom left: Principal Component Analysis (PCA) biplot showing the separation of high‐risk and low‐risk samples along the PC1 and PC2 axes. Vectors represent mutational features contributing to sample clustering. Right: Bar plot indicating the percentage of variance explained by the top five principal components (PC1–PC5) (Top row) and loading values of mutational features (C>G, TMB, T>G, C>A, Indels) for PC1 and PC2 (Bottom row). Indel, insertion and deletion variant; PC, principal component; *POLE*, DNA polymerase epsilon; TMB, tumor mutational burden.

We then analyzed 31 *POLE*mut cases with unfavorable clinical and histopathological features such as significant LVSI, negative ER/PR status, nonendometrioid histotypes, and advanced stages. Significant differences were observed between those with and without unfavorable features in the TMB (210.70 vs. 83.20, respectively; *p* = .00303) and C>G mutations (2.22 vs. 3.73; *p* = .03936). No clustering was detected in the PCA (Figure 2).

### Analysis of MC patients

Sixteen patients in group A (26.2%) were identified as MC; specifically, nine were *POLE*mut‐MMRd, three were *POLE*mut‐MMRd‐p53abn, and four were *POLE*mut‐p53abn. Clinical characteristics are shown in Table 2. Median values of the TCGA features in this group were a TMB of 218.15 Mut/Mb, 4.45% indels, 47.88% C>A, 2.44% C>G, and 0.88% T>G (Figure S3). Compared to non‐MC patients included in group A, no significant differences were found, except for the TMB (218.15 for MC cases and 123.1 for the others; *p* = .00170). At PCA, MC cases clustered along with non‐MC group A.

**TABLE 2:** Clinical and pathological characteristics of multiple‐classifier patients with endometrial cancer within group A (*N* = 16).

Characteristics	*POLE*mut‐MMRd	*POLE*mut‐MMRd‐p53abn	*POLE*mut‐p53abn
	*N* = 9	*N* = 3	*N* = 4
Age at diagnosis, mean (range), years	56 (31–70)	56 (55–56)	71 (33–88)
Histotype, No. (%)			
Endometrioid G1 and G2	4 (44.4)	0 (0.0)	0 (0.0)
Endometrioid G3	4 (44.4)	0 (0.0)	2 (50.0)
Serous	0 (0.0)	1 (33.3)	1 (25.0)
Mixed	1 (11.1)	2 (66.7)	1 (25.0)
LVSI, No. (%)			
Negative	5 (55.6)	2 (66.7)	3 (75.0)
Focal	0 (0.0)	1 (33.3)	0 (0.0)
Substantial	4 (44.4)	0 (0.0)	1 (25.0)
2009 FIGO pathological stage, No. (%)			
IA	6 (66.7)	1 (33.3)	3 (75.0)
IB	3 (33.3)	2 (66.7)	0 (0.0)
II	0 (0.0)	0 (0.0)	1 (25.0)
2023 FIGO pathological stage, No. (%)			
IAm_*POLE*mut_	9 (100.0)	3 (100.0)	4 (100.0)

Abbreviations: FIGO, International Federation of Gynecology and Obstetrics; G, grade; LVSI, lymphovascular space invasion; MMRd, mismatch repair deficient; p53abn, p53 abnormal; *POLE*, DNA polymerase epsilon.

Two patients did not display signature 10. MC7 (*POLE* mutation D368Y and MMRd) was affected by grade 3 endometrioid EC and showed 60.00% indels, 30.00% C>A, 0.00% C>G, and 0.00% T>G. She displayed COSMIC signatures 14 and 15, both related to defective DNA MMR and MSI.

MC9 (*POLE* mutation V411L and MMRd) was affected by a mixed EC, with serous (80%) and endometrioid (20%) grade 3 components and substantial LVSI. She displayed 17.77 indels, 25.00% C>A, 0.00% C>G, and 0.00% T>G. COSMIC signatures related to defective DNA MMR and MSI were identified, with a major contribution of SBS6. From a clinical perspective, she received a genetic test, which excluded defects in MMR genes.

The PCA was consistent with the signatures found because they both appeared as outliers. MC7 was found closer to the MMRd cluster, which suggested common features most in PC1; MC9 showed the highest contribution of PC2 among the *POLE* cluster. Both patients received adjuvant radiotherapy as a result of incomplete surgical staging; however, they had good outcomes, and per the last follow‐up (3.3 and 22 months, respectively), they are alive and free of disease.

### Analysis of group B

Eight patients exhibited a *POLE* nonhotspot mutation within the EDM: two were p53abn (P1 and P2), five were MMRd (P3–P7), and one was NSMP (P8) (Table 3). These patients were significantly older than those in group A (64 vs. 57 years; *p* = .029). Similar to the group A population, 62.5% of cases were endometrioid, and all cases were in 2009 and 2023 FIGO early stages.

**TABLE 3:** Clinical and pathological characteristics of group B.

Characteristics	Nonhotspot *POLE* variant
	*N* = 8
Age at diagnosis, mean (range), years	65 (52–72)
Histotype, No. (%)	
Endometrioid G1 and G2	2 (25.0)
Endometrioid G3	3 (37.5)
Serous	1 (12.5)
Mixed	2 (25.0)
LVSI, No. (%)	
Negative	3 (37.5)
Focal	2 (25.0)
Substantial	3 (37.5)
2009 FIGO pathological stage, No. (%)	
IA	5 (62.5)
IB	3 (37.5)
2023 FIGO pathological stage, No. (%)	
IA1	1 (12.5)
IA2	1 (12.5)
IIC	3 (37.5)
IIC_p53abn_	3 (37.5)

Abbreviations: FIGO, International Federation of Gynecology and Obstetrics; G, grade; LVSI, lymphovascular space invasion; MMRd, mismatch repair deficient; p53abn, p53 abnormal; *POLE*, DNA polymerase epsilon.

**FIGURE 3:**
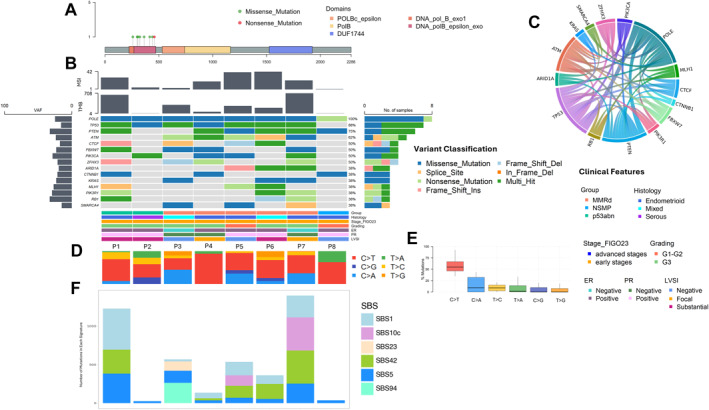
Integrative genomic and clinical characterization of patients in group B. (A) Lollipop plot representing mutation distribution in the POLE gene. Missense mutations and functional domains are reported. (B) Oncoplot displaying variants in the most frequently mutated genes and clinical features. Bar chart on the left represents the mean VAF. (C) Chord plot representing co‐mutation patterns. Width of the edges is proportional to co‐occurrence instances. (D–E) Single nucleotides substitution types. (D) Percentage of single nucleotide substitutions per sample and (E) Box plot illustrating single nucleotide substitutions frequencies for different substitution types. Points outside the whiskers represent outliers. (F) COSMIC mutational signature decomposition. Bar plot displaying the contribution of mutational signatures to each sample. Different colors represent distinct signatures. COSMIC, catalogue of somatic mutations in cancer; ER, estrogen receptor; FIGO, International Federation of Gynecology and Obstetrics; G, grade; LVSI, lymphovascular space invasion; MSI, microsatellite instability; *POLE*, DNA polymerase epsilon; PR, progesterone receptor; TMB, tumor mutational burden; VAF, variant allele frequency.

The molecular features of this group are shown in Figure 3. Seven of the eight patients had a missense *POLE* mutation, whereas one patient (P8) had a nonsense mutation (Figure 3A,B). The most commonly coaltered genes in group B were *TP53* (88%), *PTEN* (75%), and *ATM* (62%) (Figure 3B,C). These alterations are similar to those observed in group A.

The following median values of TCGA features in this group were found: a TMB of 231.85 Mut/Mb, 14.07% indels, 9.09% C>A, 0.00% C>G, and 0.00% T>G (Figure 3D,E). Compared to the group A population, significant differences were found for C>A (*p* < .001) and indels (*p* = .02) but not for the TMB (*p* = .81), C>G (*p* = .65), and T>G (*p* = .38).

P1, P3, and P5–P7 were the patients with the highest TMB among the group B subgroup (median, 302.40 Mut/Mb). Except for P3, all patients harbored a *POLE* mutation considered damaging according to different in silico tools (Table S3).

**FIGURE 4:**
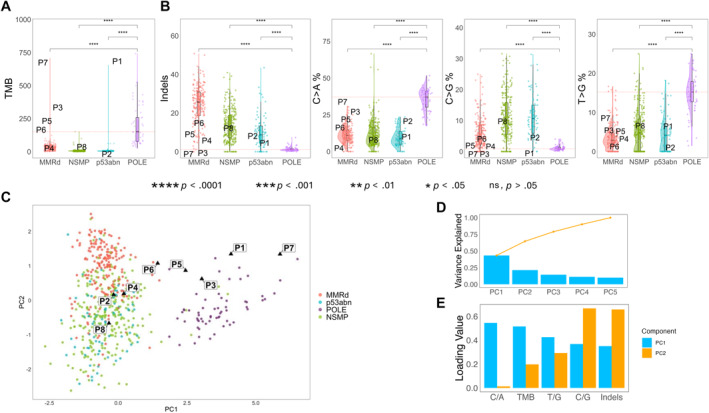
Comparison and principal component analysis of The Cancer Genome Atlas parameters across the four molecular risk groups. (A) Comparison of TMB across molecular subtypes (MMRd, NSMP, p53abn, POLE). (B) Comparison of the proportion of indels and single nucleotide substitution (C>A, C>T, T>G, T>C) across molecular risk groups. Significant differences are highlighted with asterisks. (C) Principal Component Analysis plot showing clustering of samples based on principal components (PC1 and PC2). Samples are colored by molecular risk groups, and representative sample (P1–P8) are highlighted. (D) Bar plot displaying the variance explained by the top five principal components (PC1–PC5). (E) Loading values for mutational features in PCA to PC1 and PC2. Indel, insertion and deletion variant; MMRd, mismatch repair deficient; ns, not significant; NSMP, nonspecific molecular profile; P, patient; PC, principal component; *POLE*, DNA polymerase epsilon; p53abn, p53 abnormal; TMB, tumor mutational burden.

When TCGA features of the entire MMRd group were compared with the subgroup of MMRd patients in group B (P3–P7), statistically significant differences were observed for indels (*p* = .01), T>G (*p* < .001), and TMB (*p* < .001) (Figure 4A,B). Conversely, P1 and P2 (p53abn within group B) did not exhibit any statistically significant differences compared to the broader p53abn group in terms of TCGA features. In the PCA, P1, P3, P5, and P7 displayed values greater than 2 for PC1, similar to group A (Figure 4C–E).

By the COSMIC analysis, none of the cases had signature 10a or 10b, which are commonly associated with defective *POLE* function (Figure 3F). However, in P5 and P7, a contribution of signature 10c was noticed. This signature has recently been described as associated with germline *POLD* mutations.^18^ Consistent with this finding, P7 was found to have four different somatic mutations in the *POLD1* gene.

### Additional *POLE* mutations

Besides *POLE* mutations within the EDM, all group B cases displayed additional *POLE* mutations, with different interpretations of pathogenicity (Table S4). We investigated whether the number of additional *POLE* mutations correlated with the TMB value. Our analysis revealed that patients harboring more than two *POLE* mutations exhibited a significantly higher TMB compared to those with only one mutation (*p* = .0043). However, in patients with three or more *POLE* variants of uncertain significance (VUSs), this increase in the TMB was not consistently observed (*p* = .043). A similar trend was noted in the group A population, where a significant difference in the TMB was observed between patients with two or more *POLE* mutations and those with a single mutation (*p* < .001).

## DISCUSSION

Our comprehensive analysis of prospectively clinically sequenced patients with EC revealed that only TMB values and C>G percentages differed significantly between group A patients with unfavorable clinical features and those without. Similarly, no genomic differences were observed between MC and non‐MC patients within group A, apart from TMB values. Specific *POLE* mutations, even if not considered hotspots, displayed genomic features comparable to group A. Specifically, P1, P3, P5, and P7 demonstrated greater genomic similarity to group A than to group B, where they belong.

Furthermore, the number of *POLE* VUSs increases the TMB value. In the integrated genomic characterization published by the TCGA Research Network, all 17 patients with EC displaying an ultramutant phenotype had mutations in the EDM of *POLE*, with a high prevalence (76%) of hotspot mutations P286R and V411L.^3^ León‐Castillo et al. expanded the list of *POLE* hotspot variants in 2020.^4^ Variants outside this list had different prognostic behavior compared to hotspot *POLE* mutations (hazard ratio [HR], 3.4; 95% CI, 1.5–7.6; log‐rank *p* < .01), which are not fully deciphered yet.^19^, ^20^ This study presents a comprehensive overview of pathogenic and nonpathogenic *POLE* mutations in a large cohort of EC cases, which highlights shared clinical and molecular characteristics. Although it does not alter the current criteria for classifying patients with EC as *POLE*mut, this study examines genomic and clinical features that, with extended follow‐up data, may lead to reclassification in the future. This is particularly relevant because recent evidence in solid tumors has shown that patients with pathogenic *POLE* mutations, even outside the EDM, may benefit from immune checkpoint inhibitors.^21^ Recently, a subanalysis of the RUBY trial demonstrated an advantage in MMR‐proficient/p53abn tumors of the addition of dostarlimab to standard chemotherapy, which showed a reduction in the risk of disease progression or death by 37% (HR, 0.63; 95% CI, 0.44–0.91).^22^ This is in contrast with the results of a subanalysis of the GARNET trial, in which the p53 subgroup showed lower progression‐free survival and duration of response compared to MMRd, except for those patients who displayed a high TMB.^23^ ^,^ ^24^ ^.^ This condition is similar to the one of P1, who harbored the *POLE* P436S mutation and eight other *POLE* mutations of unknown significance outside the EDM. This *POLE* mutation has already been reported in colorectal cancer as being similar to known *POLE* hotspot mutations.^25^ Consistently, P1 displayed a TMB of 650.7 Mut/Mb. It could be argued that the high TMB can be caused by MSI‐high (unstable sites, 27.1%). In MMRd patients, in fact, additional (secondary) *POLE* mutations, including hotspot mutations, may emerge. There is conflicting evidence regarding whether *POLE* mutations in MMRd cases act as primary drivers of hypermutation by occurring first or arise as secondary events because of the preexisting high mutational burden.^26^, ^27^, ^28^ In P1, the ultramutated phenotype (TMB, >100 Mut/Mb), in silico tool prediction, and TCGA features are consistent with a *POLE* hotspot instead of a p53abn case. Furthermore, a genetic blood test in this patient ruled out Lynch syndrome.

P5 had the *POLE* T278K mutation, which was previously linked to ultramutated features and MSI‐high status in both EC and colorectal cancer. This *POLE* mutation was also shown to cause secondary mutations in MMR genes.

Our results are in line with previous evidence that demonstrated that the TMB was significantly higher in different types of tumors with *POLE* hotspot mutations plus other nonhotspot *POLE* mutations (*p* < .001).^29^ Furthermore, the presence of several VUSs in *POL‐*family genes has been shown to exhibit a prolonged overall survival.^30^

It must be acknowledged that median values of the genomic features were calculated according to the output of a tumor‐only multigene assay, which may differ from the data obtained via whole‐genome sequencing, such as that used in TCGA. Moreover, the rate of *POLE*mut cases was slightly higher (10.2%) than that expected and reported in the literature (7%–10%). This could be an indirect effect of routine adoption of CGP rather than target solutions or Sanger sequencing, and is consistent with recent data on the rates of *POLE*mut patients.^31^

The main limitations of this study include the low prevalence of *POLE* mutations, which constrained the analysis, and the limited follow‐up duration, which prevented us from drawing definitive conclusions regarding the true prognostic significance of *POLE* mutations within group B that exhibit genomic features resembling those of group A. Finally, the absence of an external cohort to validate our findings prevents the generalizing of results.

Although more data with an adequate follow‐up are maturing, the present findings must be considered hypothesis generating, which indicates that a more comprehensive analysis of both group A and B cases could help in the future to uncover potentially unexpected prognostic behaviors.

## AUTHOR CONTRIBUTIONS

**Camilla Nero**: Conceptualization; writing—original draft; writing—review and editing. **Rita Trozzi**: Conceptualization; writing—original draft. **Federica Persiani**: Formal analysis; visualization; software. **Simone Rossi**: Formal analysis; visualization; software. **Luca Mastrantoni**: Conceptualization; writing—original draft. **Simona Duranti**: Conceptualization; writing—review and editing. **Floriana Camarda**: Conceptualization. **Ilenia Marino**: Data curation; project administration. **Luciano Giacò**: Software; data curation; visualization. **Tina Pasciuto**: Methodology; data curation; software. **Maria De Bonis**: Investigation. **Martina Rinelli**: Investigation. **Emanuele Perrone**: Conceptualization. **Flavia Giacomini**: Project administration; data curation. **Domenica Lorusso**: Supervision; writing—review and editing. **Alessia Piermattei**: Investigation. **Gianfranco Zannoni**: Supervision; writing—review and editing. **Francesco Fanfani**: Supervision; writing—review and editing. **Giovanni Scambia**: Supervision; writing—review and editing. **Angelo Minucci**: Investigation; conceptualization.

## CONFLICT OF INTEREST STATEMENT

Camilla Nero reports travel support from Merck Sharp and Dohme, Illumina, Menarini, and AstraZeneca, ​and honoraria from Veeva, GlaxoSmithKline, Merck Sharp and Dohme, AstraZeneca, Illumina, and Guardant Health. Domenica Lorusso reports research funding from Clovis, GlaxoSmithKline, Merck Sharp and Dohme, ImmunoGen, Novartis, Seagen, AstraZeneca, Genmab, Alkermes, and Corcept; consulting for Clovis, Seagen, Novartis, GlaxoSmithKline, ImmunoGen, Merck Sharp and Dohme, Corcept, Novocure, Roche Health Solutions, PharmaMar, Genmab, AstraZeneca, and Daiichi Sankyo; travel support from Menarini, Merck Sharp and Dohme, AstraZeneca, and GlaxoSmithKline; personal interests with AstraZeneca, Clovis, GlaxoSmithKline, PharmaMar, and Merck Sharp and Dohme; and financial interests with Clovis, Genmab, GlaxoSmithKline, and Merck Sharp and Dohme. Francesco Fanfani reports research funding from Clovis, GlaxoSmithKline, Merck Sharp and Dohme, and PharmaMar, and personal and financial interests with GlaxoSmithKline, Merck Sharp and Dohme, Sysmex, and Stryker. Giovanni Scambia reports research funding from Merck Sharp and Dohme, honoraria from Clovis, and consulting for Tesaro and Johnson & Johnson. The other authors declare no conflicts of interest.

## Supporting information

Supplementary Material S1

Figure S1

Figure S2

Figure S3

## Data Availability

The data sets generated and analyzed during the current study are not publicly available because of ethical reasons but are available from the corresponding author on reasonable request.
