# Supplementary material for: POLE mutations in endometrial carcinoma: Clinical and genomic landscape from a large prospective single‐center cohort
Source: Cancer. 2025 Jan 25;131(3):e35731. doi: 10.1002/cncr.35731 (PMC11771542; doi:10.1002/cncr.35731)
Supplement: Supplementary file 1 — Supplementary Material S1 [file CNCR-131-0-s003.docx]

*Immunohistochemistry*

Immunohistochemistry positive signals for MMR proteins (MSH2, MSH6, MLH1 and MSH2) and p53 were detected with the labeled streptavidin–biotin peroxidase detection system using the UltraView Universal DAB Detection Kit and the OptiView DAB IHC Detection Kit on the BenchMark ULTRA instrument (Ventana), respectively. MMR deficiency was defined by the loss of expression of one or more MMR proteins (MSH2, MSH6, MLH1 and MSH2) in all tumor cell nuclei, with stromal/lymphocyte staining and non-neoplastic endometrial glands serving as positive internal controls. Abnormal p53 expression is declared if over 10% of tumoral surface presented strong positive nuclear staining in 80-100% of tumour cell nuclei (overexpression), complete absence of staining with positive internal control or cytoplasmic staining (Singh N, Piskorz AM, Bosse T, et al. p53 immunohistochemistry is an accurate surrogate for TP53 mutational analysis in endometrial carcinoma biopsies. J Pathol. 2020;250(3):336-45.)

*Genomic data analysis*

The assay adopted, TruSight Oncology 500 high-throughput panel (TSO500HT, Illumina) analyzes both DNA and RNA detecting SNVs, *indels* and copy number variations (CNVs) in 523 genes, as well as known and unknown fusions and splicing variants in 55 genes. Additionally, it assesses genomic "signatures" like microsatellite instability (MSI) and TMB, indicating the total number of somatic mutations in the sequenced genome. TMB was considered high if ≥ 10 Mutation/Megabase (Mut/Mb). (Supplementary)

The Illumina Software Dragen TSO500 HT v2.5 processed raw sequencing data.

Samples were sequenced with a mean depth of > 500×. The minimum coverage accepted for variant calling was 100× on 90% of sequenced targeted regions and at least 250× on hotspot regions. VCF files containing the identified variants were converted into Mutation Annotation Format (MAF) and functionally annotated using Ensembl Variant Effect Predictor (VEP) and vcf2maf (<https://github.com/mskcc/vcf2maf.git>) using default parameters. Then, MAF files were annotated with the OncoKB annotator. Results were restricted to genes with a VAF cutoff of 0.05 and Moderate/High impact.

Indels and SNVs with an allelic frequency above 0.04% in gnomAD v2 or 1000 Genomes Project were excluded and only mutations classified as "Oncogenic" or "Likely Oncogenic", according to OncoKB , were considered for the analysis. Oncogenic variants belonging to the levels IA, IB, IIC according to the AMP/ASCO/CAP recommendations and to the HGVS nomenclature (www.hgvs.org/mutnomen), were also reported.

*Exclusion criteria for TMB calculation*

The following criteria were applied to exclude variants from the tumor mutational burden (TMB) calculation:

- Non-PASS Variants: variants not passing the quality control filters.
- Mitochondrial Variants: Variants located in mitochondrial DNA.
- MNVs (Multi-Nucleotide Variants): Multi-nucleotide variations.
- Depth Threshold: Variants that do not meet the minimum sequencing depth threshold.
- Variant Allele Threshold: Variants that do not meet the minimum variant allele frequency threshold.
- Eligibility of Regions: Variant located outside the pre-deined eligible genomic regions.
- Tumor Driver Mutations: Variants with a population allele count >= 50 are treated as tumor driver mutations and excluded.

Germline variants are excluded from TMB calculation.

The determination of germline variants was performed using a combination of a database and a proxy filter:

Population Database Criteria:

- Variants with a population allele count >= 10 in either the 1000 Genomes Project or gnomAD database are marked as germline
- For MNVs, if all their component small variants are marked as germline, the MNVs themselves are also marked as germline.

Proxy Filter:

- The proxy filter assesses the variant allele frequencies of surrounding variants.
- A specific variant is marked as germline if the majority of its surrounding variants with similar VAF are classified as germline.

This stringent filtering ensures that only somatic mutations, pertinent to tumor’s mutational landscape, are included in the TMB calculation.

| **Supplementary table 1** Clinical and pathological characteristics of 596 endometrial cancer included in the study | | | | | |
| --- | --- | --- | --- | --- | --- |
| Characteristics | All cases | POLE mut | MMRd | p53 abn | NSMP |
|  | n=596 | n=61 | n=205 | n=77 | n=253 |
| Mean age at diagnosis, years | 62 (27-89) | 55 (31-88) | 63 (33-88) | 67 (36-87) | 62 (27-89) |
| Histotype |  |  |  |  |  |
| Endometrioid G1-2 | 372 (62.4) | 27 (44.3) | 124 (60.5) | 4 (5.2) | 217 (85.8) |
| Endometrioid G3 | 117 (19.6) | 20 (32.8) | 64 (31.2) | 5 (6.5) | 28 (11.1) |
| Serous | 46 (7.7) | 2 (3.3) | 2 (1.0) | 41 (53.2) | 1 (0.4) |
| Clear cells | 9 (1.5) | 3 (4.9) | 4 (2.0) | 1 (1.3) | 1 (0.4) |
| Mixed | 34 (5.7) | 6 (9.8) | 8 (3.9) | 16 (20.8) | 4 (1.6) |
| Dedifferentiated | 6 (1.0) | 1 (1.6) | 3 (1.5) | 1 (1.3) | 1 (0.4) |
| Carcinosarcoma | 12 (2.0) | 2 (3.3) | 0 (0.0) | 9 (11.7) | 1 (0.4) |
| LVSI |  |  |  |  |  |
| Not available * | 14 (2.3) | 0 (0.0) | 4 (2.0) | 7 (9.1) | 3 (1.2) |
| Negative | 369 (61.9) | 41 (67.2) | 110 (53.7) | 35 (45.5) | 183 (72.3) |
| Focal | 87 (14.6) | 6 (9.8) | 36 (17.6) | 11 (14.3) | 34 (13.4) |
| Substantial | 126 (21.1) | 14 (23.0) | 55 (26.8) | 24 (31.2) | 33 (13.0) |
| 2009 Figo pathological stage |  |  |  |  |  |
| IA | 277 (46.5) | 36 (59.0) | 80 (39.0) | 27 (35.1) | 134 (53.0) |
| IB | 171 (28.7) | 17 (27.9) | 67 (32.7) | 16 (20.8) | 71 (28.1) |
| II | 60 (10.1) | 4 (6.6) | 27 (13.2) | 7 (9.1) | 22 (8.7) |
| IIIA | 11 (1.8) | 1 (1.6) | 4 (2.0) | 3 (3.9) | 3 (1.2) |
| IIIB | 6 (1.0) | 0 (0.0) | 3 (1.5) | 2 (2.6) | 1 (0.4) |
| IIIC1 | 40 (6.7) | 2 (3.3) | 12 (5.9) | 7 (9.1) | 19 (7.5) |
| IIIC2 | 4 (0.7) | 0 (0.0) | 2 (1.0) | 2 (2.6) | 0 (0.0) |
| IVA | 1 (0.2) | 0 (0.0) | 0 (0.0) | 1 (1.3) | 0 (0.0) |
| IVB | 26 (4.4) | 1 (1.6) | 10 (4.9) | 12 (15.6) | 3 (1.2) |
| 2023 Figo pathological stage |  |  |  |  |  |
| IA1 | 32 (5.4) | 0 (0.0) | 13 (6.3) | 0 (0.0) | 19 (7.5) |
| IA2 | 150 (25.2) | 0 (0.0) | 49 (23.9) | 0 (0.0) | 101 (39.9) |
| IA3 | 2 (0.3) | 0 (0.0) | 1 (0.5) | 0 (0.0) | 1 (0.4) |
| IAm_POLEmut_ | 57 (9.6) | 57 (93.4) | 0 (0.0) | 0 (0.0) | 0 (0.0) |
| IB | 74 (12.4) | 0 (0.0) | 23 (11.2) | 0 (0.0) | 51 (20.2) |
| IIA | 25 (4.2) | 0 (0.0) | 11 (5.4) | 0 (0.0) | 14 (5.5) |
| IIB | 25 (4.2) | 0 (0.0) | 14 (6.8) | 0 (0.0) | 11 (4.3) |
| IIC | 82 (13.8) | 0 (0.0) | 54 (26.3) | 0 (0.0) | 28 (11.1) |
| IIC_p53abn_ | 60 (10.1) | 0 (0.0) | 8 (3.9) | 50 (64.9) | 2 (0.8) |
| IIIA1 | 11 (1.8) | 1 (1.6) | 5 (2.4) | 2 (2.6) | 3 (1.2) |
| IIIA2 | 2 (0.3) | 0 (0.0) | 1 (0.5) | 1 (1.3) | 0 (0.0) |
| IIIB1 | 5 (0.8) | 0 (0.0) | 2 (1.0) | 2 (2.6) | 1 (0.4) |
| IIIB2 | 2 (0.3) | 0 (0.0) | 0 (0.0) | 1 (1.3) | 1 (0.4) |
| IIIC1i | 21 (3.5) | 2 (3.3) | 1 (0.5) | 2 (2.6) | 16 (6.3) |
| IIIC1ii | 20 (3.4) | 0 (0.0) | 12 (5.9) | 5 (6.5) | 3 (1.2) |
| IIIC2ii | 3 (0.5) | 0 (0.0) | 1 (0.5) | 2 (2.6) | 0 (0.0) |
| IVA | 2 (0.3) | 0 (0.0) | 1 (0.5) | 1 (1.3) | 0 (0.0) |
| IVB | 16 (2.7) | 0 (0.0) | 6 (2.9) | 9 (11.7) | 1 (0.4) |
| IVC | 7 (1.2) | 1 (1.6) | 3 (1.5) | 2 (2.6) | 1 (0.4) |
| Results are presented as n (%) except where indicated. MMRd: MisMatch Repair deficient. NSMP: No Specific Molecular Profile. LVSI: Lymph Vascular Space Invasion. FIGO: International Federation of Gynecology and Obstetrics * In twelve cases only biopsy was performed | | | | | |

| **Supplementary Table 2**: Types of POLE mutations within the Group A | | |
| --- | --- | --- |
| **POLE mutation** | **Percentage** | **Number of patients** |
| P286R | 59.0 % | 36 |
| V411L | 19.7 % | 12 |
| A456P | 6.6 % | 4 |
| S297F | 6.6 % | 4 |
| S459F | 4.9 % | 3 |
| D368Y | 1.6 % | 1 |
| L424F | 1.6 % | 1 |
| M444K | 1.6 % | 1 |
| P286S | 1.6 % | 1 |

| **Supplementary Table 3:** Molecular characteristics of Group B patients | | | | | | | | |  |
| --- | --- | --- | --- | --- | --- | --- | --- | --- | --- |
| Patient | MSI (unstable sites) | TMB (muts/Mb) | Group | Protein change | Variant allele frequency | SIFT | POLYPHEN | REVEL | MetaRNN |
| P-1 | UNSTABLE (27.1%) | HIGH (650.7) | P53 | P436S | 31,9% | Deleterious (0) | Probably damaging (1) | Uncertain (0.524) | Pathogenic Moderate (0.8822) |
| P-2 | STABLE (4.9%) | LOW (3.9) | P53 | D287E | 37.7% | Deleterious (0) | Probably damaging (0.996) | Benign Moderate (0.286) | Benign Strong (0.04419) |
| P-3 | STABLE (5.2%) | HIGH (301.4) | MMRd | A428T | 16.8% | Toletared (0.31) | Benign (0.017) | Benign Moderate (0.33) | Uncertain, (0.5159) |
| P-4 | UNSTABLE (18.3%) | HIGH (57.4) | MMRd | T278M | 31.0% | Deleterious (0) | Probably damaging (0.999) | Uncertain (0.619) | Pathogenic Moderate (0.8704) |
| P-5 | UNSTABLE (37.8%) | HIGH (268.5) | MMRd | T278K | 30.0% | Deleterious (0) | Probably damaging (0.999) | Uncertain (0.666) | Pathogenic Moderate (0.8926) |
| P-6 | UNSTABLE (40.2%) | HIGH (197.6) | MMRd | N363K | 5.8% | Deleterious (0) | Probably damaging (1) | Pathogenic Supporting (0.735) | Pathogenic Strong (0.9759) |
| P-7 | STABLE (0.1%) | HIGH (714.5) | MMRd | S297Y | 30% | Deleterious (0) | Probably damaging (1) | Pathogenic Supporting (0.792) | Pathogenic Moderate (0.9158) |
| P-8 | STABLE (18.1%) | MEDIUM (5.5) | NSMP | Y458 | 24.1% | N.A. | N.A. | Benign Moderate (0.286) | Benign Supporting (0.4242) |

N.A. not available

| **Supplementary Table 4**: Variants of unknows significance in the Group B | | | | | | | |
| --- | --- | --- | --- | --- | --- | --- | --- |
| Patient | Protein change | Variant Classification | REVEL | SIFT | PolyPhen | CLINVAR | ONCO KB |
| P-1 | R1579C | Missense mutation | Benign Supporting (0.412) | Deleterious (0) | Probably_damaging (0.959) | Uncertain_significance | Unknown |
| P-1 | R1386W | Missense mutation | Benign Supporting (0.409) | Deleterious (0) | Probably_damaging (0.984) | Uncertain_significance | Unknown |
| P-1 | R1286C | Missense mutation | Uncertain (0.487) | Deleterious (0) | Probably_damaging (0.985) | Uncertain_significance | Unknown |
| P-1 | T1052M | Missense mutation | Uncertain (0.515) | Deleterious (0) | Probably_damaging (0.957) | Uncertain_significance | Unknown |
| P-1 | F990I | Missense mutation | Uncertain (0.545) | Deleterious (0) | Probably_damaging (0.996) | Not reported | Unknown |
| P-1 | R759C | Missense mutation | Uncertain (0.682) | Deleterious (0) | Probably_damaging (0.999) | Uncertain_significance | Unknown |
| P-1 | R222C | Missense mutation | Benign Supporting (0.469) | Deleterious (0) | Probably_damaging (0.991) | Uncertain_significance,conflicting_interpretations_of_pathogenicity | Unknown |
| P-3 | E2140D | Missense mutation | Benign Strong (0.016) | Tolerated (0.67) | Benign (0.003) | Not reported | Unknown |
| P-3 | D2115Y | Missense mutation | Pathogenic Supporting (0.7) | Deleterious (0) | Probably_damaging (0.993) | Not reported | Unknown |
| P-3 | I234V | Missense mutation | Benign Moderate (0.36) | deleterious(0.01) | Probably_damaging (0.986) | Uncertain_significance | Unknown |
| P-4 | p.? | Splice region |  |  |  | Uncertain_significance,benign,likely_benign | Unknown |
| P-4 | T528M | Missense mutation | Uncertain (0.486) | Deleterious (0.01) | Probably_damaging (0.943) | Uncertain_significance,conflicting_interpretations_of_pathogenicity | Unknown |
| P-5 | A1375V | Missense mutation | Benign Strong (0.041) | Tolerated (0.07) | Benign (0) | Uncertain_significance | Unknown |
| P-5 | p.? | Splice region |  |  |  | Uncertain_significance,benign,likely_benign | Unknown |
| P-6 | S2062N | Missense mutation | Benign Strong (0.045) | Tolerated (0.49) | Benign (0) | Uncertain_significance | Unknown |
| P-6 | p.? | Splice region |  |  |  | Not reported | Unknown |
| P-6 | p.? | Splice region |  |  |  | Uncertain_significance,benign,likely_benign | Unknown |
| P-7 | L1455I | Missense mutation | Benign Moderate (0.169) | Tolerated (0.06) | Benign (0.027) | Not reported | Unknown |
| P-7 | P1226H | Missense mutation | Benign Moderate (0.161) | Tolerated (0.06) | Benign (0.012) | Not reported | Unknown |
| P-7 | D287E | Missense mutation | Benign Moderate (0.259) | Deleterious (0) | Probably_damaging (0.996) | Uncertain_significance,conflicting_interpretations_of_pathogenicity,benign/likely_benign | Unknown |

**Supplementary Figure Legend**

Supplementary Figure 1: Consort diagram

Supplementary Figure 2: Oncoprint of the cohort

Supplementary Figure 3: Multiclassifier patients among Group A
