# Supplementary material for: POLE mutations in endometrial carcinoma: Clinical and genomic landscape from a large prospective single‐center cohort
Source: Cancer. 2025 Jan 25;131(3):e35731. doi: 10.1002/cncr.35731 (PMC11771542; doi:10.1002/cncr.35731)
Supplement: Supplementary file 2 — Figure S1 [file CNCR-131-0-s004.pdf]

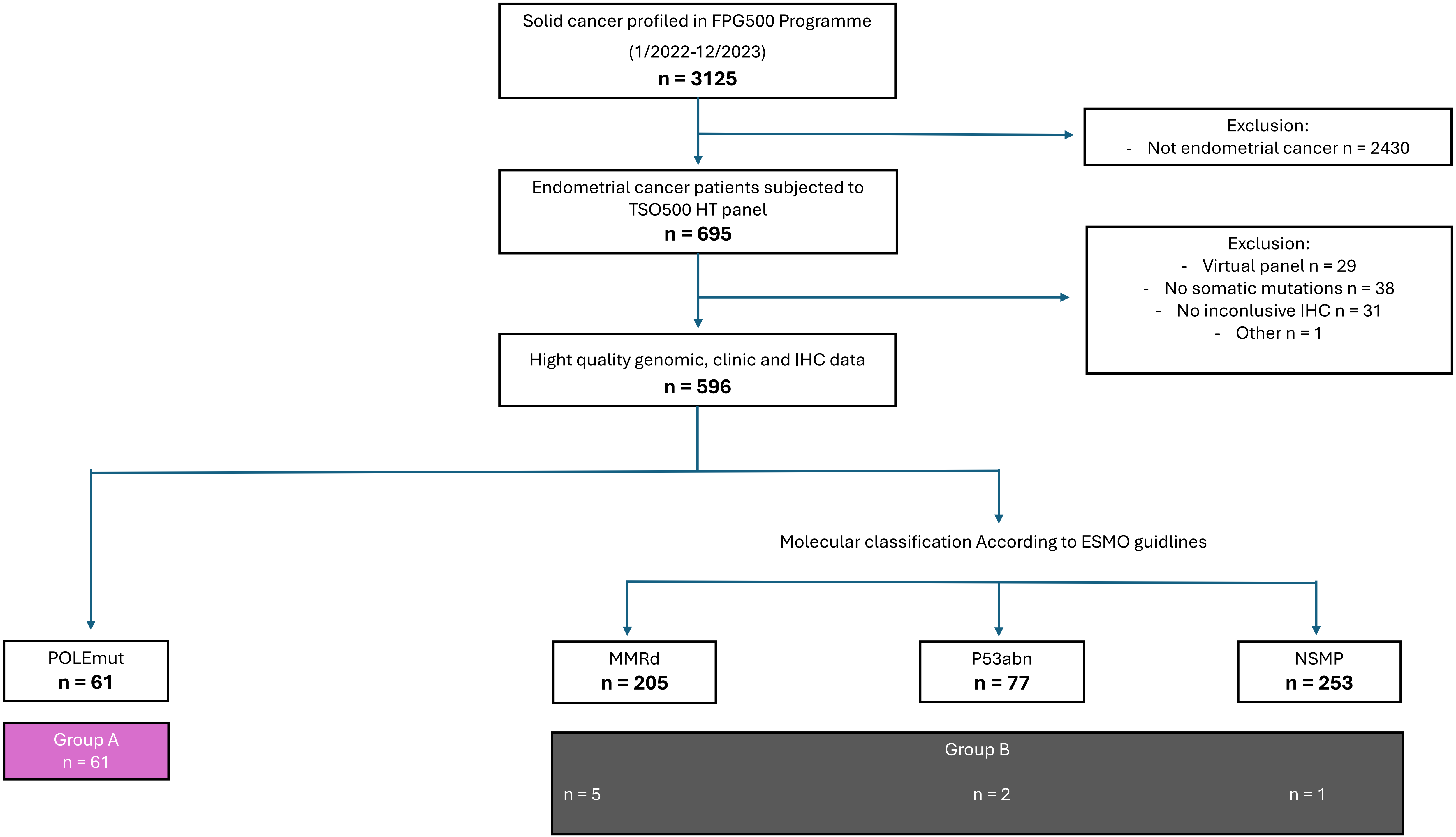

Solid cancer profiled in FPG500 Programme
(1/2022-12/2023)
n = 3125
Exclusion:
- Not endometrial cancer n = 2430
Endometrial cancer patients subjected to TSO500 HT panel
n = 695
Exclusion:
- Virtual panel n = 29
- No somatic mutations n = 38
- No inconlusive IHC n = 31
- Other n = 1
Hight quality genomic, clinic and IHC data
n = 596
Molecular classification According to ESMO guidlines
POLEmut
n = 61
MMRd
n = 205
P53abn
n = 77
NSMP
n = 253
Group A
n = 61
Group B
n = 5
n = 2
n = 1
