# Supplementary material for: POLE mutations in endometrial carcinoma: Clinical and genomic landscape from a large prospective single‐center cohort
Source: Cancer. 2025 Jan 25;131(3):e35731. doi: 10.1002/cncr.35731 (PMC11771542; doi:10.1002/cncr.35731)
Supplement: Supplementary file 3 — Figure S2 [file CNCR-131-0-s002.pdf]

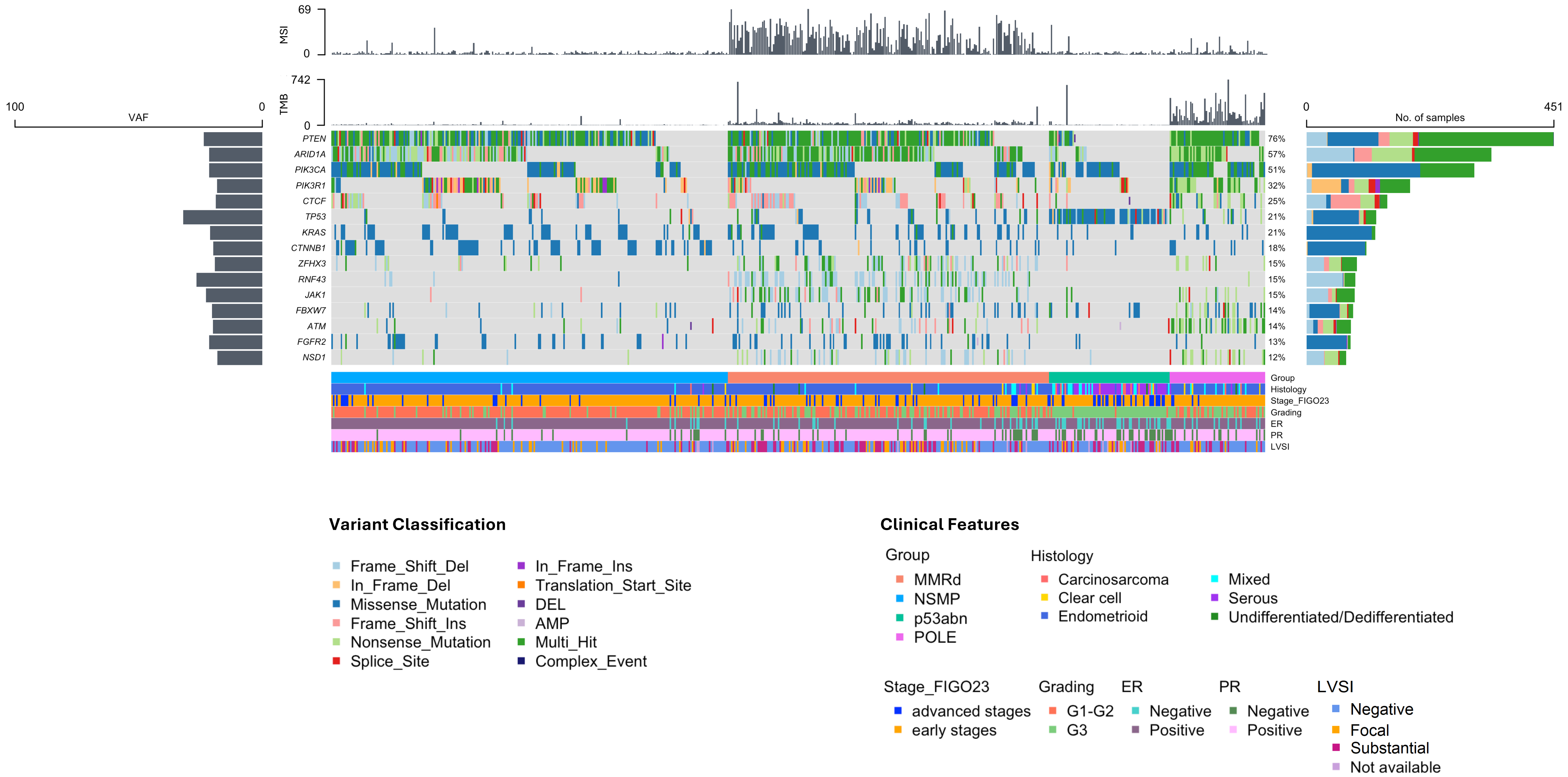
MSI
69
0
TMB
742
0
100
VAF
0
0
No. of samples
451
PTEN 76%
ARID1A 57%
PIK3CA 51%
PIK3R1 32%
CTCF 25%
TP53 21%
KRAS 21%
CTNNB1 18%
ZFHX3 15%
RNF43 15%
JAK1 15%
FBXW7 14%
ATM 14%
FGFR2 13%
NSD1 12%
Group
Histology
Stage_FIGO23
Grading
ER
PR
LVSI
Variant Classification
Frame_Shift_Del
In_Frame_Del
Missense_Mutation
Frame_Shift_Ins
Nonsense_Mutation
Splice_Site
In_Frame_Ins
Translation_Start_Site
DEL
AMP
Multi_Hit
Complex_Event
Clinical Features
Group
MMRd
NSMP
p53abn
POLE
Histology
Carcinosarcoma
Clear cell
Endometrioid
Mixed
Serous
Undifferentiated/Dedifferentiated
Stage_FIGO23
advanced stages
early stages
Grading
G1-G2
G3
ER
Negative
Positive
PR
Negative
Positive
LVSI
Negative
Focal
Substantial
Not available
