# Supplementary figures and images for: POLE mutations in endometrial carcinoma: Clinical and genomic landscape from a large prospective single‐center cohort
Source: Cancer. 2025 Jan 25;131(3):e35731. doi: 10.1002/cncr.35731 (PMC11771542; doi:10.1002/cncr.35731)

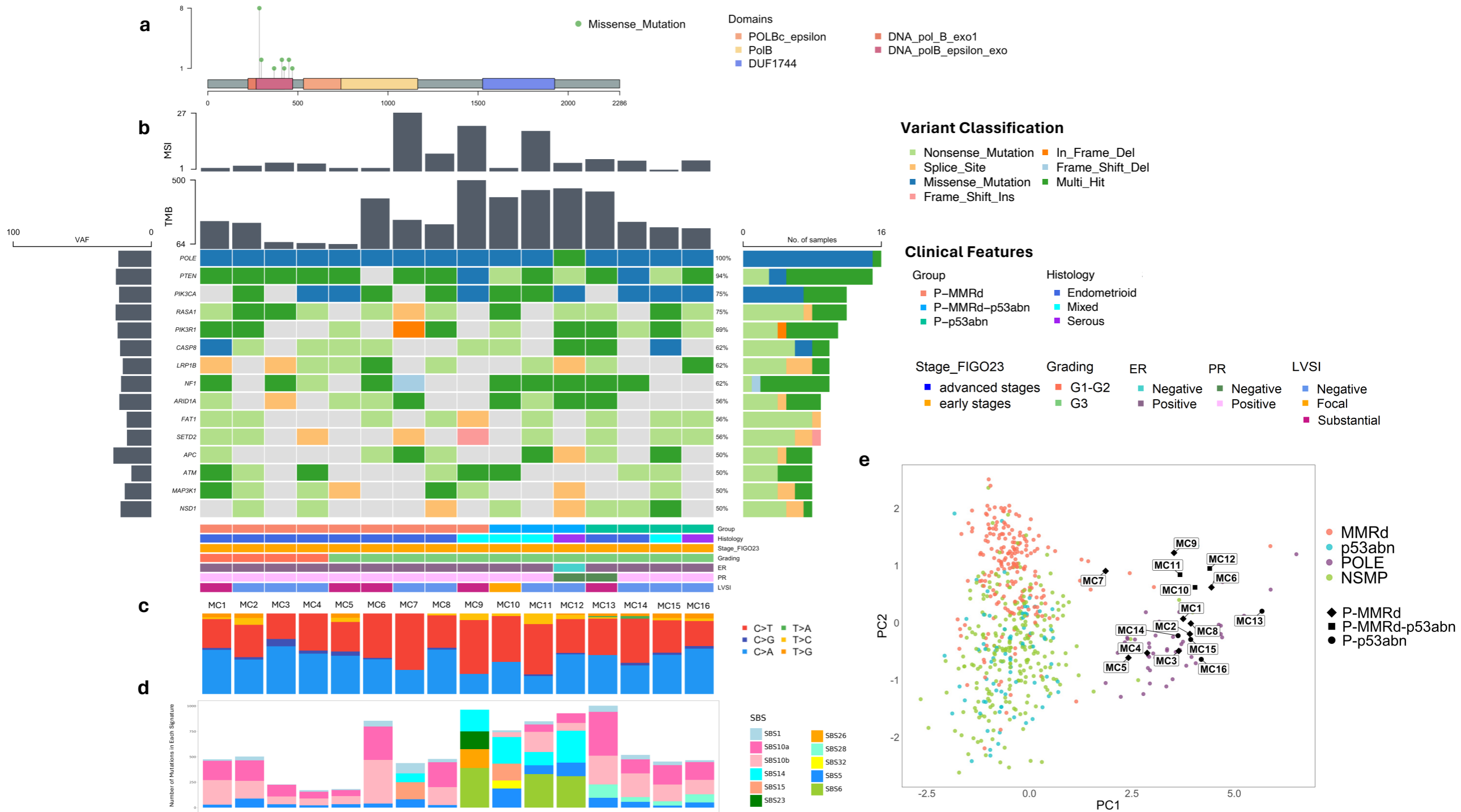

Supplement: Supplementary file 4 — Figure S3 [file CNCR-131-0-s001.pdf]
